# A Top-Down Procedure for Synthesizing Calcium Carbonate-Enriched Chitosan from Shrimp Shell Wastes

**DOI:** 10.3390/gels8110742

**Published:** 2022-11-15

**Authors:** Andreea Miron, Andrei Sarbu, Anamaria Zaharia, Teodor Sandu, Horia Iovu, Radu Claudiu Fierascu, Ana-Lorena Neagu, Anita-Laura Chiriac, Tanta-Verona Iordache

**Affiliations:** 1 Advanced Polymer Materials and Polymer Recycling Group, National Institute for Research & Development in Chemistry and Petrochemistry ICECHIM, Spl. Independentei 202, 6th District, 060021 Bucharest, Romania; 2 Advanced Polymer Materials Group, University POLITEHNICA of Bucharest,1-7 Gh. Polizu Street, 011061 Bucharest, Romania

**Keywords:** shrimp shell waste, calcium-carbonate-enriched chitosan, crystallinity, thermal stability, rheology

## Abstract

Chitosan is used in medicine, pharmaceuticals, cosmetics, agriculture, water treatment, and food due to its superior biocompatibility and biodegradability. Nevertheless, the complex and relatively expensive extraction costs hamper its exploitation and, implicitly, the recycling of marine waste, the most abundant source of chitosan. In the spirit of developing environmental-friendly and cost-effective procedures, the present study describes one method worth consideration to deliver calcium-carbonate-enriched chitosan from shrimp shell waste, which proposes to maintain the native minerals in the structure of chitin in order to improve the thermal stability and processability of chitosan. Therefore, a synthesis protocol was developed starting from an optimized deacetylation procedure using commercial chitin. The ultimate chitosan product from shrimp shells, containing native calcium carbonate, was further compared to commercial chitosan and chitosan synthesized from commercial chitin. Finally, the collected data during the study pointed out that the prospected method succeeded in delivering calcium-carbonate-enriched chitosan with high deacetylation degree (approximately 75%), low molecular weight (Mn ≈ 10.000 g/ mol), a crystallinity above 59 calculated in the (020) plane, high thermal stability (maximum decomposition temperature over 300 °C), and constant viscosity on a wide range of share rates (quasi-Newtonian behavior), becoming a viable candidate for future chitosan-based materials that can expand the application horizon.

## 1. Introduction

Since the beginning, the polymer industry has relied exclusively on environmentally harmful petroleum derivatives, which slowly shifted several decades ago toward biologically sourced polymers, i.e., renewable and/or biodegradable polymers. However, the need for sustainable and biodegradable materials that are renewable and commercially available, at the same time, has been accelerated in recent years, by the dynamics of human society. For this reason, natural polymers have gained popularity over their synthetic counterparts with poor biocompatibility and biodegradability. For instance, natural polymers such as polysaccharides (starch, cellulose, lignin, and chitin) are used in various applications, including wastewater treatment, tissue engineering, and controlled drug release [1,2].

Chitin, a natural amino-polymer with a long chain polymer of β-(1-4)-N-acetyl-D-glucosamine, is a structural biopolymer with a similar function to that of collagen in higher animals and that of cellulose in plants. This biopolymer naturally occurs in the exoskeleton of insects, arthropods, and crustaceans, being the second most abundant biopolymer after cellulose [3,4]. Although used in India, Korea, China, and Egypt since ancient times, research on chitin began in the 19th century [5,6]. According to Crini [6], chitin can be found in three types of arrangements, named α, β, and γ. Type α is the most robust and resistant to physical and chemical aggression and the most widespread in nature. It presents an antiparallel orientation of the molecules, thus strengthening itself through hydrogen bonds [6,7]. Chitin is traditionally extracted from the marine shells, by washing, crushing, and deproteinization of shell flakes, and it may be quickly turned into chitosan using biological or chemical processes, the latter benefiting from cost-efficiency and versatility [1,7].

Chitosan was first obtained by C. Rouget by boiling chitin in a concentrated alkaline solution, then observing that the resulting compound was soluble in organic acids. Later on, F. Hoppe-Seiler confirmed that chitosan is the deacetylated form of chitin, giving it its current name [3,5]. Thus, chitosan is a natural linear polyaminosaccharide, insoluble in organic solvents and only soluble in weakly acidic aqueous medium (pH < 6.5) forming a gel. Chitosan presents a series of advantages that make it an ideal candidate for many applications, such as the food, cosmetics, and pharmaceutical industry, but also as an antimicrobial agent in clinical applications. It belongs to a new class of biomaterials with low toxicity, extremely good biocompatibility, and adsorption properties [8].

Nevertheless, the route from seashell wastes to chitosan is somewhat complex and time consuming, implying the following main steps [9,10,11]: (i) demineralization—minerals removal, (ii) deproteinization—proteins removal, and finally (iii) deacetylation—removing acetyl pendant groups to form hydroxyl groups. 

Traditional demineralization of seashells waste is achieved by extracting calcium carbonate and other phosphorous-based minerals in a mild acidic medium of HCl 0.1 M–1 M, at 25–120 °C, for 1 h to 2 days [12,13].

The following deproteinization stage is difficult due to the breaking of the chemical bonds between chitin and proteins. Complete removal of proteins is necessary, especially for food and biomedical applications, because an important percentage of the population can manifest allergies to crustaceans, mainly due to proteins [7]. Therefore, the chemical methods of deproteinization have been intensively addressed in the specialized literature [11,14,15]. For this purpose, a wide range of reagents have been used, including NaOH, KOH, NaHCO_3_, and Ca(OH)_2_. Yet, NaOH is the preferred reagent due to its low cost and accessibility. Using this latter route, the shells are generally ground and treated with NaOH (0.125 M–5 M) at temperatures between 24 °C and 160 °C for a few minutes or even a few days. The use of NaOH inevitably results in a partial deacetylation, thus leading to lower molecular weights of the biopolymer [15].

In the next step of deacetylation, the most frequent treatment relies on using strong alkaline solutions and high temperatures. Here as well, NaOH is commonly used. Among the factors influencing the deacetylation degree, it is worth mentioning NaOH concentration, reaction time, and temperature [11,13,16]. Chitin deacetylation can be performed either homogeneously or heterogeneously. The heterogeneous approach involves treating chitin with a hot NaOH solution, yielding a chitosan with a degree of deacetylation ranging from 85 to 99%; this procedure aims for uneven deacetylation throughout the polymer chain. The homogeneous approach involves dispersing chitin in a concentrated NaOH solution at room temperature for 3 h or more, followed by dissolving in crushed ice at around 0 °C. This technique yields chitosan with a degree of deacetylation ranging between 48 and 55 % [11].

Although about 2000 t of chitosan is produced each year from residues of shrimp or crab shells, its proper exploitation and recycling remain challenging due to the high costs [7,17,18]. In order to extend the recycling and valorization degree of such wastes, the research for other applications has now intensified [19]. However, this would also mean that innovative solutions for synthesizing chitosan at lower costs should be prospected [18]. 

Furthermore, it is well known that one of the main drawbacks of chitosan is its mechanical and thermal stability, especially in gels. Several authors have presented solutions for enhancing the mechanical and thermal stability of chitosan by adding silver nanowires or carbon nanotubes [20,21]; inorganic fillers such as CaCO_3_, silica, and zinc [19,22]; or blending with other (bio)polymers [23,24,25].

However, one cheap and environmental-friendly solution proposed in this work for delivering chitosan with enhanced thermal stability refers to maintaining the native minerals in the chitin/chitosan structure. In other words, the proposed method refers to by-passing the demineralization step in chitin extraction, to yield calcium-carbonate-enriched chitosan. By doing so, the complex and important mineralization process performed by marine organisms, namely that of storing CO_2_ from seawater, is also valued [26]. In this respect, this study presents a top-down procedure for synthesizing calcium-carbonate- enriched chitosan, starting from optimizing the process of commercial chitin deacetylation to yield chitosan with low molecular weight and high deacetylation degree, followed by application of this process to chitin obtained from shrimp shells waste by direct deproteinization. To the best of our knowledge, such a top-down procedure for chitosan synthesis from shrimp shells waste has never been reported in the literature.

One of the advantages of the proposed synthesis technology described in this study, derived from bypassing the demineralization step, refers to the lower consumption of energy and time, and implicitly reduced preparation costs. As a parallel, a study of He et al. described the preparation of chitosan-based composites enriched with CaCO_3_, which included additional stages for preparing the enriched chitosan [27]. CaCO_3_ was first synthesized, by colloidal crystallization from supersaturated solution, followed by filtration and drying. In the next step, chitosan was dissolved in an aqueous acetic acid solution and used as a dispersion phase for the inorganic phase. Therefore, the additional phases of the process employed in this case involve higher costs by consumption of energy and resources. Another major advantage resulting from applying the technology refers to delivering chitosan with improved thermal properties and processability, compared to chitosan prepared by other methods, which include the demineralization step.

## 2. Results and Discussion

### 2.1. Optimization of Chitosan Synthesis from Commercial Chitin

The degree of deacetylation, DD, differentiates chitin from chitosan. Chitosan is obtained when a DD above 50% is attained and the product is soluble in acidic aqueous solutions. During the deacetylation step, a depolymerization reaction also takes place, which leads to a decrease in the product molecular weight [28,29].

For this reason, the first stage of this research targeted the optimization of the chitosan synthesis process, trials beginning with commercial chitin-derived chitosan (CCHC), and ending by establishing the optimal synthesis procedure for delivering chitosan with low molecular weight and high deacetylation degree. In this respect, various chitosan samples were synthesised from commercial chitin, by changing various parameters of the procedure (reaction time and number of deacetylation cycles). Three sample types of commercial chitin-derived chitosan were produced at various synthesis times either in a direct batch (CCHCt, where t = 4 and 6 h and represents the reaction time for the first deacetylation cycle) or in 2 steps [(CCHC4 (t′), where t′ = 2 and represents the reaction time for the second deacetylation cycle] in order to determine the optimal reaction time and procedure. Hence, for one sample, chitosan obtained after 4 h (CCHC4) was dried and subjected to another alkaline treatment for 2 h.

#### 2.1.1. FTIR Structural Analysis of CCHC Series

**Figure 1:**
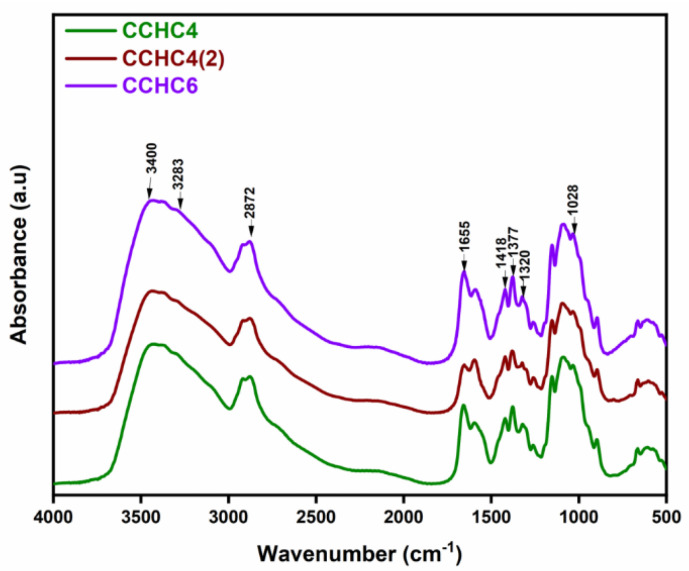
FTIR spectra of the obtained commercial chitin-derived chitosan samples at different deacetylation times.

The FTIR spectra provided information on the chemical structure of the chitosan samples. According to Figure 1, all samples showed similarities regarding the main characteristic bands. The stretching vibrations of the N–H and O–H bonds, as well as the vibrations of the intramolecular hydrogen bonds, can be distinguished in the 3400–3283 cm^−1^ region. The band from 2872 cm^−1^ was typical for the –CH bonds stretching vibration, characteristic of polysaccharides such as xylan and glucan [9,10]. The N-acetyl groups contained distinctive bands in the range of 1655 cm^−1^ for the tensile vibration of the C=O bonds in amide I, and at 1320 cm^−1^ for the stretching vibration of C=O in amide III. The bands at approximately 1418 cm^−1^ and 1377 cm^−1^ belonged to the stretching vibration of the –CH_2_ bond and deformation of the –CH_3_, while the stretching vibrations of the O–C bonds were highlighted by the bands recorded at 1022 cm^−1^ and 1028 cm^−1^. Furthermore, the synthesized chitosan samples showed all the characteristic bands described in the literature [30,31]. In addition, the number of deacetylation cycles and the reaction time of the process did not bring significant changes in the structure of chitosan obtained from commercial chitin.

#### 2.1.2. Deacetylation Degree and Molecular Weight Distribution of CCHC Series

In order to decide which deacetylation procedure was best suited, the different samples of commercial chitin-derived chitosan (CCHC), the DD values were first calculated using the titration method. According to the values given in Table 1, the DDs were significantly different, indicating that the DD values depended highly on the synthesis parameters employed. The results obtained based on the variation in chitosan pH as a function of 0.1 M HCl solution volume are shown in Figure S1a–c (Supporting Information). Thus, it was observed that DD increased with the increase in the reaction time from 4 h to 6 h, reaching a maximum of 78.19% after 6 h in one cycle, which was closest to the DD of the commercial chitosan (CC). Meanwhile, the introduction of an additional deacetylation cycle proved to be helpful for intermediary DD values, but overall, the process was not quite economic.

Further on, GPC measurements, in terms of average number molecular weight (Mn) and polydispersity index (PI), helped in evaluating the potential of two employed deacetylation procedures, i.e., single-cycle or two-cycle (values given in Table 1). On top of the DD, the molecular weight of chitosan is one of the most important characteristics as it considerably affects its physicochemical properties [32]. Generally, the difference in the molecular weight of chitosan may be due to the different sources of origin, but also to a heterogenous deacetylation degree, as obtained in this study as well. Moreover, several factors in the chitosan synthesis process (chitin concentration, alkali concentration, the high temperature, and time of reaction) may also influence its molecular weight [33]. One may notice from Table 1 and Figure S2 (Supporting Information) that for all synthesized chitosan samples, two populations, with different molecular weights and PI, appeared. The first population exhibited higher Mn and PI values compared to the second population. Yet, all the chitosan samples presented a dominant peak, with Mn of same order of magnitude, which accounted for over 90% of the analyzed sample, while the CC presented only one peak in the same range. Having the sample of CCHC4 as a reference, it seemed that the deacetylation approach in two steps (CCHC4(2)) led to an increased Mn, compared to the one performed in one step at 6 h, CCHC6. Interestingly, the sample closest to CC was CCHC4(2) with almost 98% of chitosan of the same molecular weight. However, this same sample also registered the highest Mn and a lower DD value compared to CCHC6.

### 2.2. Synthesis and Characterization of Chitin and Chitosan from Shrimp Shells Waste

**Scheme 1:**
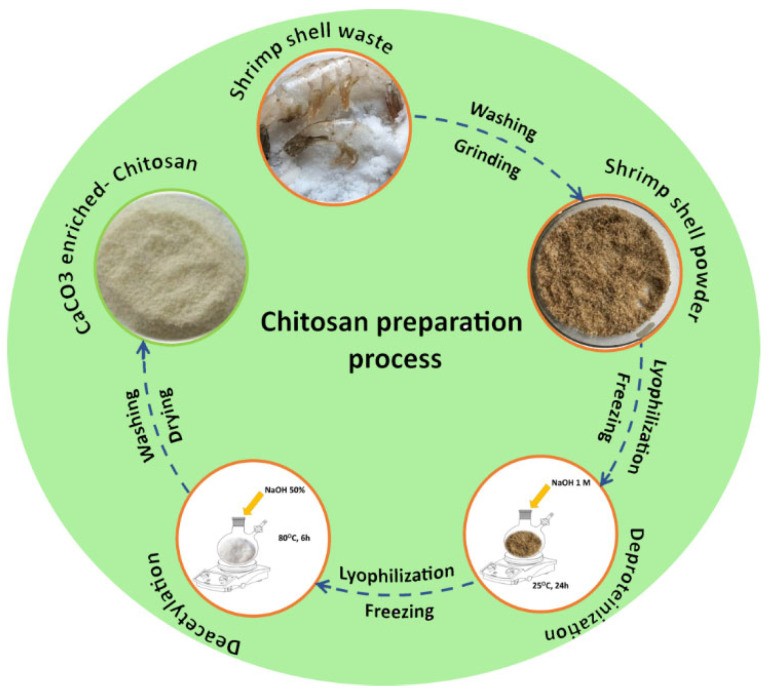
Preparation process of calcium-carbonate-enriched chitosan from shrimp shells waste.

As a consequence of the data recorded after chitin deacetylation, in terms of structure, molecular weight distribution, and DD results, it was concluded that for a higher degree of deacetylation and low molecular weights, the ideal synthesis time for chitosan was at least 6 h in one cycle. Therefore, this optimized procedure was also applied for preparing calcium-carbonate-enriched chitosan from shrimp shells as illustrated in Scheme 1. In order to have a better understanding of this process, the chitosan series of commercial chitosan (CC), chitosan prepared from commercial chitin (CCHC6), and chitosan from shrimp shells (CCHSH) were compared in each step.

#### 2.2.1. FTIR Spectroscopy of CCHSH and Intermediaries

**Figure 2:**
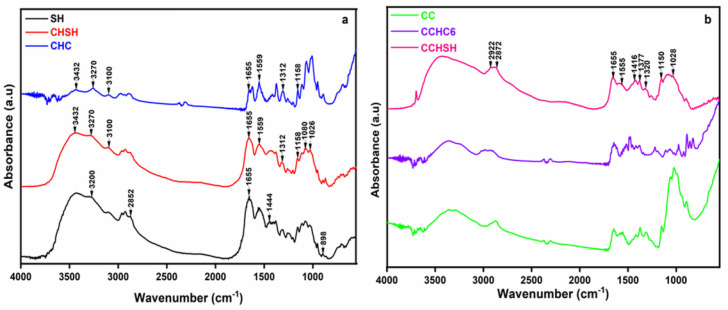
FTIR spectra of the SH, CHSH, and CHC (**a**) and of CC, CCHC6, and CCHSH (**b**) samples.

Figure 2 depicts a comparison between biopolymers isolated from shrimp shells (chitin and chitosan) and their commercially available counterparts, with the aim of highlighting the characteristic groups.

The FTIR spectrum of shrimp shells (SH, Figure 2a) showed the occurrence of different functional groups on its surface. Broad peaks were detected in the 3500–3200 cm^−1^ region, which were attributed to the vibrations of the O–H bond, from intramolecular water molecules and to the N-H group. The band detected at 2852 cm^−1^ corresponded to the stretching vibrations C-H of aliphatic hydrocarbons, while the value of 1655 cm^−1^ was attributed to the stretching vibrations of C=O (from amide I). In addition to the former analyzed spectrum of chitosan obtained from commercial chitin, the bands recorded in this case at 1444 cm^−1^ and 898 cm^−1^ were attributed to the stretching and bending vibrations of CaCO_3_ [34]. By analyzing the FTIR spectrum of chitin (CHSH) obtained from shrimp shell (SH) waste, the presence of O-H and N-H groups at 3432 cm^−1^ and 3270–3100 cm^−1^, respectively, may also be observed. Again, the most interesting bands in the chitin spectrum were those appearing at 1655, 1559, and 1312 cm^−1^, assigned to the characteristic vibrations of amides I, II, and III, respectively. Several bands were also spotted at 1158, 1080, and 1026 cm^−1^, belonging to C–O–C bonds in the chitin structure. When comparing commercial chitin (CHC) to chitin from shrimp shells (CHSH), the intensity of bands in the area 3500–3000 cm^−1^ was lower for CHC. Nevertheless, the chitin extracted from SH exhibited all the above-mentioned typical bands of chitin, as well as the presence of the amide I peaks at 1627 and 1656 cm^−1^, indicating an α-chitin, but with a spectral resemblance with that of SH, due to the fact that calcium carbonate was still present in the structure, leading to various intramolecular bonding.

Similarities were observed between laboratory-synthesized chitosan (CCHC6 and CCHSH) samples and commercial chitosan (CC) (Figure 2b). Absorption bands at 2922 and 2872 cm^−1^ can be attributed to the symmetrical and asymmetrical stretching of the C–H bond, typical bands of polysaccharides [31]. In the case of the CCHSH sample, a higher intensity of the bands in the 2924-2872 cm^−1^ region was observed due to the C–H groups that also attested the presence of calcium carbonate [35]. The residual N-acetyl groups were confirmed by the bands at 1655 cm^−1^ (C=O group from amide I) and 1320 cm^−1^ (C–N bond from amide III), respectively. Unlike chitin, the 1555 cm^−1^ band, characteristic of the N–H bending vibration group in amide II, was no longer observed, which was probably caused by the high deacetylation degree of the produced chitosan, compared to the commercial chitosan. The –CH_2_ bending and the –CH_3_ symmetrical deformations were confirmed by the bands at 1416 cm^−1^ and 1377 cm^−1^, respectively. The absorption band at 1150 cm^−1^ was attributed to the asymmetric stretching of the C–O–C bond, while the bands from 1059 cm^−1^ to 1028 cm^−1^ corresponded to the C–O vibrations.

As the samples of chitosan were of animal origin, possible contamination with glycosaminoglycans (GAGs, another type of polysaccharide [31]) was also perused. GAGs can be identified by the appearance of very strong bands around 1260–1270 cm^−1^, corresponding to the sulphate groups. As the CCHSH chitosan shows only a weak signal at 1260 cm^−1^ (Figure 2b), the possibility of GAGs contamination of the obtained chitosan was, thus, excluded. 

#### 2.2.2. Deacetylation Degree and Molecular Weight Distribution of CCHSH

The tests performed on the chitosan series CC, CCHC6, and CCHSH, shown in Figure S1c–e (titration plot, Supplemental Information) and Table 1, revealed very small differences between the DD values calculated for CC, CCHC6, and CCHSH. The value of DD registered for CCHSH was higher compared to the one of CCHC4(2), indicating that the procedure of deacetylation in one cycle of 6 h was indeed optimum for delivering calcium-carbonate-enriched chitosan from shrimp shells with high DD.

The molecular weight distribution for the CC, CCHC6, and CCHSH samples is shown comparatively in Figure S2 (Supporting Information). According to the summarized value of Mn and PI in Table 1, the final product CCHSH presented higher Mn values only for the first peak, i.e., 1655 × 10^6^ g/mol, compared to CC and CCHC6, which ultimately resulted in a slight lower solubility of the synthesized chitosan in acetic acid solution (solubility in 10% acetic acid solution vs. 5% for CC and CCHC). This behavior of the CCHSH sample was somewhat expected, given the presence of calcium carbonate in the structure, a fact confirmed by FTIR and further XRD analyses. However, CCHSH also attained intriguingly low PI values for both populations (meaning 6.19 and 1.39, respectively) as compared to the other chitosan samples in this series, which indicates that chitosan macromolecular chains are of similar length; a PI value equal to 1 would mean that all the macromolecular chains are of the same size [36]. This result means that the approached deacetylation processes, which is essentially an heterogenous process, was better controlled from the point of view of heterogeneity by the presence of calcium carbonate. Another noteworthy aspect of the CCHSH molecular weight distribution is the fact that the main population was represented by the second peak, with over 70% of chitosan in the sample, but with slight lower Mn values compared to the counterparts (i.e., 9.059 × 10^3^). Although the percentage of CCHSH with low molecular weight was smaller than that represented by the first peak in CC and CCHC6 samples (70% < 92% < 100%), the approached deacetylation procedure in one cycle for 6 h was able to deliver calcium-carbonate-enriched chitosan from shrimp shells, having low molecular weight and high deacetylation degree, as initially proposed.

#### 2.2.3. XRD Patterns of CCHSH and Intermediaries

**Figure 3:**
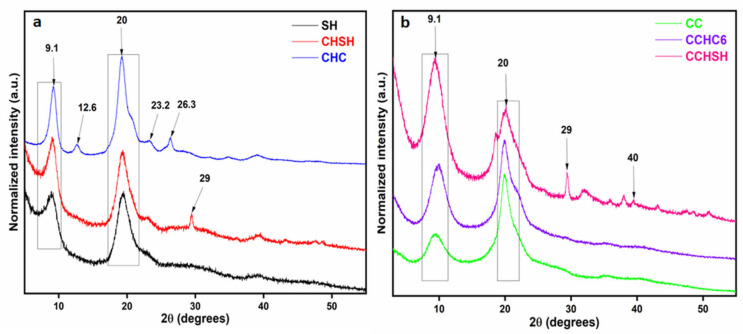
XRD patterns for the SH, CHSH, and CHC (**a**) and CC, CCHC6, and CCHSH (**b**) series.

X-ray diffraction (XRD) is one of the most versatile and widely used techniques for investigating the physicochemical properties of biopolymers and their crystallinity, which is why the XRD profiles of shrimp shell waste, chitin, and chitosan samples were evaluated here as well. In Figure 3a, the diffractograms of shrimp shells (SH) of chitin extracted from shrimp shell (CHSH) waste and of commercial chitin (CHC) are compared, while in Figure 3b, the series of chitosan samples CC, CCHC6, and CCHSH are investigated.

CHC presented high-intensity diffraction peaks at 9.1° and 20.0°, corresponding to planes 020 and 110 (Figure 3a). In particular, three other peaks of lower intensity were observed at 12.6°, 23.2°, and 26.3°, which diffract on planes 021, 130, and 013, indicating the structure of α-type chitin [37]. On the same graph, SH and CHSH exhibited similar peaks characteristic to α-type chitin, distinguished at 9.08°, 19.3°, and 23.3° (corresponding to planes 020, 110, and 130), with the mention that other peaks appeared at 29.0° in plane 104 and 40.8° in plane 116, corresponding to CaCO_3_ [18]. For SH, the presence of CaCO_3_ was barely observed due to the high the content of proteins (30–40%) [38]. 

In Figure 3b, the crystalline structure of chitosan samples is compared. Peaks at 2θ = 9.1° and 2θ = 20.0° could be observed for all chitosan samples, indicating the ordered crystalline structure of chitosan. The absence of the peak at approximately 12.6° from the 021 plane, characteristic of the chitin pattern, was an indication for the efficient deacetylation process. However, the XRD patterns of CCHSH revealed the presence of CaCO_3_ through the characteristic peaks at 2θ values of 40.0° and 29.0°, as also acknowledged for the intermediaries CHSH and SH. 

The crystallinity indexes determined in chitin/chitosan planes (020) and (110) are centralized in Table 2. The values of both indexes revealed that commercial chitin, CHC, presented a higher crystallinity compared to CHSH, synthesized in the laboratory, which may be due to higher contents of water in SH and thereof. Although the samples were dried to constant weight (by freeze-drying), water may remain bonded to CaCO_3_ and, thereafter, affect the evaluation of samples crystallinity [39]. In the chitosan series, however, CrI_020_ indicated the highest crystallinity value for CCHSH, which, by some authors, may be due to CaCO_3_ [40]. Therefore, it can also be presumed that the remanent water in SH, CHSH, and CCHSH may be a consequence of the CaCO_3_ presence, and their actual crystallinity content may differ as a function of their humidity [39].

#### 2.2.4. Thermogravimetric Profiles (TGA/DTG) of CCHSH and Intermediaries

**Figure 4:**
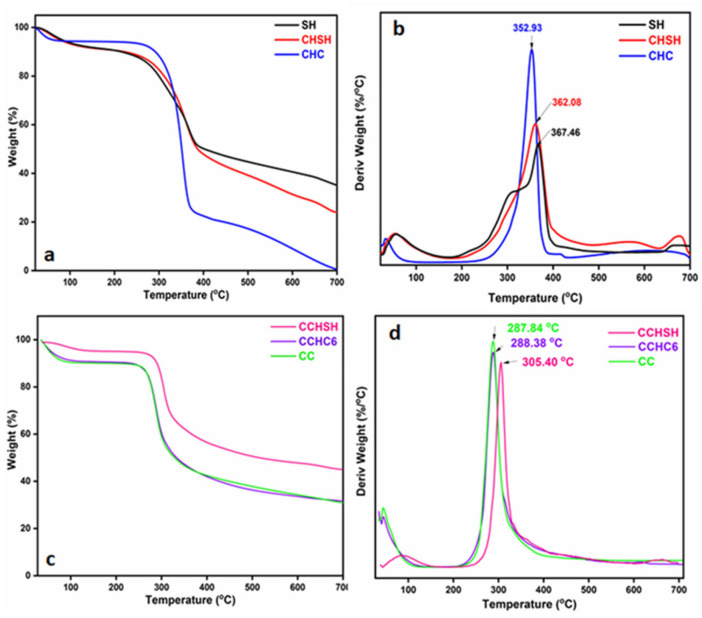
TGA (**a**) and DTG (**b**) curves for SH, CHSH, and CHC samples; TGA (**c**) and DTG (**d**) curves for CC, CCHC6, and CCHSH samples.

In order to provide evidence for the fact that the proposed synthesis approach for CCHSH is worth consideration and that keeping the native calcium carbonate in the structure can enhance the thermal stability of chitosan, Thermogravimetric Analysis (TGA) and Differential Thermogravimetry (DTG) were performed. Figure 4a,c depict the TGA curves for shrimp shells, chitin, and chitosan, whereas Figure 3b,d show the corresponding DTG curves of the three analyzed samples. In Table 2, the maximum degradation temperatures and total mass loss of all the investigated samples are summarized for convenience.

Shrimp shells exhibited a multi-stage degradation pattern with a specific mass loss of 65.57% [31,35], as presented in Figure 4a,b. Evaporation of water occurred in the range of 30–150 °C and the breakdown of proteins and lipids in the carcasses occurred within the second stage of degradation, which took place between 150 and 400 °C (shoulder). A similar trend was observed for CHSH as well, but without the shoulder associated with protein decomposition. It can be observed that for SH and CHSH, a higher water content was lost (approximately 10% vs. 6% for CHC) at a higher maximum decomposition temperature (specific for bounded water molecules) (Table 2), thus confirming the presumptions from XRD analysis. During the second stage of degradation, a considerable mass loss was recorded for all the samples exhibited in Figure 3a,b, which was attributed to the decomposition of acetylated units of chitin, followed by the polysaccharide chain fragmentation [25]. The final stage of thermal degradation, between 600 and 700 °C, corresponded to the decomposition of CaCO_3_ into CaO and CO_2_ and was only observed for SH (weakly) and CHSH. 

Further on, the chitosan degradation occurred within two main stages, as depicted in Figure 4c,d. The first stage between 30 and 150 °C was again attributed to water loss, in which case CCHSH presented a higher maximum decomposition temperature. As also observed for SH and CHSH, the CCHSH lost the intramolecular water at higher temperatures compared to their homologues, indicating a higher thermal stability that can be due to the presence of CaCO_3_. The mass losses in this step ranged from 5 to 10%, depending on the type of chitosan. In this case, the lowest mass loss was attained by CCHSH, and the highest corresponded to CC, thus explaining the crystallinity differences observed by XRD analyses. In the second degradation stage, the degradation of the biopolymer occurred at temperatures between 200 and 400 °C, with mass losses ranging from 34 to 45%. In this step as well, the lowest mass loss was registered for CCHSH, which again pointed toward a higher thermal stability compared to the other chitosan samples in the series. This presumption was sustained by the maximum decomposition temperature of 305.4 °C attained for CCHSH. The narrow decomposition interval of CCHSH (250–400 °C) is highly linked with the homogeneity of the sample, which, in this case, confirms the data obtained by GPC for the PI. Last but not least, a third step of degradation between 600 and 700 °C is observed only for CCHSH, similar to the one observed for SH and CHSH, thus confirming that the CaCO_3_ remains in the structure even after deacetylation (as confirmed by FTIR and XRD) and may be the key factor contributing to the increased homogeneity and thermal stability [19].

#### 2.2.5. N_2_ Adsorption/Desorption Measurements and Morphology of CCHSH and Intermediates

**Figure 5:**
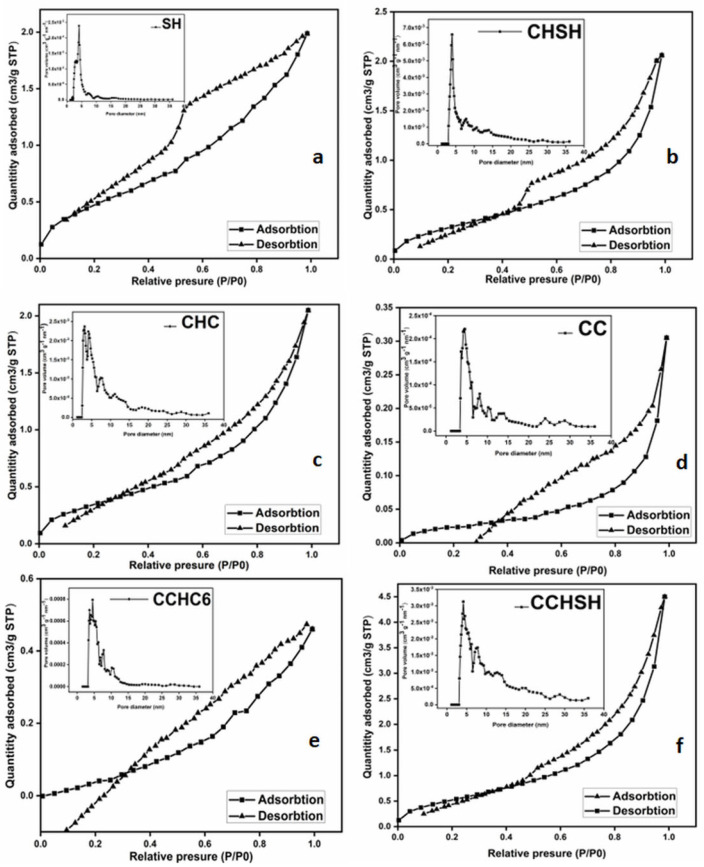
N_2_ adsorption/desorption isotherms of (**a**) SH, (**b**) CHSH, (**c**) CHC, (**d**) CC, (**e**) CCHC6, and (**f**) CCHSH materials. Inset: Pore distribution diagram (0–40 nm range).

The porosity of materials was determined via BET textural analysis. Porous materials are typically classified in terms of pore size based on gas sorption data and IUPAC standards for pore size categorization and gas adsorption/desorption isotherms representing the link between porosity and adsorption. According to the IUPAC classification, materials from this investigation belong to the category IV mesoporous domain [38]. The empirical categorization of hysteresis loops used by the IUPAC is based on an earlier Boer classification. Figure 4 depicts the IUPAC-classified adsorption/desorption curves of the produced materials. Samples SH, CHSH, and CCHSH (Figure 5a,b, and f, respectively) demonstrate an H1-type hysteresis loop associated with porous materials composed of well-defined cylindrical pore channels or almost homogenous spheres, while an H3-type hysteresis loop with slotted holes can be observed for commercial chitin (CHC, Figure 5c) and the other two variants of chitosan (CC, CCHC6, Figure 5d, and e, respectively) [41]. Table 3 summarizes the material BET surface area and pore characteristics. From this evaluation, an increase in the BET surface area can be observed for CHSH, as compared to SH (which is most likely due to protein removal [42]), but also compared to commercial chitin, CHC. Together with the BET surface area, the pore surface area of CHSH was also about two times greater relative to the CHC, for which the explanation may be linked to the freeze-drying procedure applied for all the laboratory-synthesized samples. In this sense, higher BET surface area and pore surface area were also observed when comparing CCHSH or CCHC6 to CC, which seemed to be more compact. Although the maximum pore diameter of all analyzed samples varied very little, the micropore volume of CHSH and CCHSH indicated that other factors may influence the increase in the BET surface area and pore surface area (also visible in the inset of Figure 5a–f, variation in pore volume versus pore diameter). In this respect, other studies have indicated that deproteinization and the following deacetylation reaction may increase the pore surface area and, implicitly, the pore volume [42]. However, in this thermal analysis, a key factor may also be responsible for the increase in the pore volume and diameter. The ability of CaCO_3_ to bond additional water molecules leads to larger pores and channels, as compared to CC and CHC. This new observation may explain the good solubility of CCHSH in acetic acid solutions compared to expectations.

#### 2.2.6. Rheological Properties of CCHSH

Chitosan is widely used for preparing blends or gel materials for various applications from medicine to water treatment, which is why the rheological behavior of chitosan received great importance and has been evaluated until now by several authors [43,44]. In almost all studied cases, the shear thinning behavior of chitosan solutions was found to be more pronounced as a function of the chitosan concentration, pH, temperature, surfactant, the DD, and presence of salt [45].

**Figure 6:**
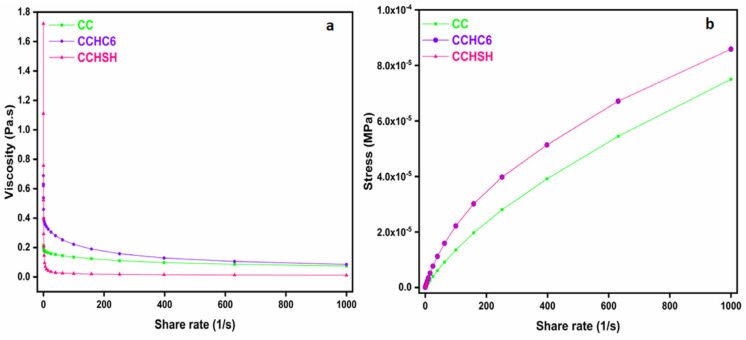
Viscosity (**a**) and stress (**b**) analysis of commercial chitosan (CC), chitosan obtained from commercial chitin (CCHC6), and chitosan obtained from shrimp shells (CCHSH).

In this study, the investigated factor of influence was CaCO_3_ at the limit of solubility, i.e., 1 g of chitosan/mL of solution. Figure 6 reveals the dependence of viscosity and stress as a function of the applied share rate for CC, CCHC6, and CCHSH. For all the chitosan samples, the shear stress increased with increasing shears, while the viscosity decreased significantly at low shear rates, after which it remained essentially constant at higher shear rates [43,46], particularly for CCHSH. Although similar, only CC and CCHC6 solutions exhibited a clear non-Newtonian pseudoplastic behavior [40], while CCHSH presented a quasi-Newtonian behavior, due to the CaCO_3_ presence. A similar behavior was reported by Martinez et al. [47] when studying the influence of salt addition on the rheology of concentrated chitosan solutions. Compared to other investigated factors, such as temperature, pH, or decreased chitosan concentration, the addition of a salt was most effective in reducing the viscosity of chitosan, acting as a lubricant [47]. Therefore, maintaining the native CaCO_3_ in chitosan not only improved the thermal stability but also helped in reducing the viscosity at high shear rates. This ultimate improvement is proof to the fact that using calcium-carbonate-enriched chitosan may represent the future for more environmental-friendly and cost-effective chitosan-based materials.

## 3. Conclusions

The first objective of the study referred to establishing an optimal procedure for synthesizing chitosan from commercial chitin, having high deacetylation degree and low molecular weight for processability reasons, which served as input for the following deacetylation procedure of chitin derived from shrimp shell waste. According to the preliminary deacetylation study, the optimal conditions to obtain low-molecular-weight chitosan (5.729 × 10^5^ g/mol) with high deacetylation degree (78.19%) corresponded to the procedure performed in one deacetylation cycle for 6 h. Further on, this procedure was successfully applied to obtain calcium-carbonate-enriched chitosan from shrimp shell wastes with equivalent or even improved physical properties relative to commercial chitosan and chitosan derived from commercial chitin. In this respect, DD, Mn, and PI of calcium-carbonate-enriched chitosan were found to be in the acceptable range as initially proposed, with values of 75.38%, 9.059 × 10^3^ g/mol, and 1.39 (for the main population of 70%), respectively. The presence of CaCO_3_ in the enriched chitosan sample was confirmed by XRD patterns, in which case the crystallinity degree was 59.42 (according to the calculated CrI in 020 plane), in which case the ability of CaCO_3_ to bond supplementary water molecules yielded denser pore structures during drying. Further thermal analysis highlighted the higher thermal stability of calcium-carbonate-enriched chitosan compared to commercial chitosan and the one derived from commercial chitin, while the rheology measurements indicated a quasi-Newtonian behavior of calcium-carbonate-enriched chitosan concentrated solution; both improvements were linked to the presence of calcium carbonate in the sample. Therefore, the preservation of calcium carbonate in the structure of chitosan not only reduces the synthesis price but also endows the final product with superior features, worth considering for developing future chitosan-based materials.

## 4. Materials and Methods

### 4.1. Materials

Commercial chitosan (CC, Sigma Aldrich-St Louis, MO, USA) and chitin (CHC, Glentham Fife Science—Corsham, UK) were used without further purification. Shrimp shells (*Litopenaeus vannamei*) (SH) obtained from a local supermarket were subjected to a deproteinization process with 1 M sodium hydroxide solution (NaOH, Sigma Aldrich—St Louis, MO, USA) in order to obtain chitin from shrimp shell waste (CHSH). Sodium chloride (NaCl, ROTH, 99.8% purity—Karlsruhe, Germany) was used without purification, as a pre-treatment agent for the shrimp shells. The preparation of chitosan from commercial chitin (CCHC) and from shrimp shells (CCHSH) involved deacetylation of the corresponding chitin by its reaction with sodium hydroxide 50% (solution prepared by dissolving NaOH, from CHIMREACTIV SRL (Bucharest, Romania), in distilled water).

Hydrochloric acid 0.1 M (HCl, Fluka—Seelze, Germany) was used to determine the degree of deacetylation of chitosan by titration. Glacial acetic acid (AcOH, CHIMREACTIV SRL—Bucharest, Romania) was used to prepare the 5% aqueous solutions of chitosan and ultrapure water (Milli Q Water Purification System) was used as the mobile phase for molecular weight measurements.

### 4.2. Synthesis of Chitosan

The procedure for the synthesis of calcium-carbonate-enriched-chitosan envisioned a top-down procedure: optimization of deacetylation process starting from commercial chitin (CHC) to yield chitosan with low molecular weights and high deacetylation degrees; isolation of chitin from shrimp shells waste bypassing demineralization; applying the optimized deacetylation procedure to obtain chitosan from shrimp-derived chitin; comparison of physicochemical properties with those of commercial chitosan and chitosan derived from commercial chitin.

#### 4.2.1. Synthesis of Chitosan from Commercial Chitin

In order to prepare chitosan, commercial chitin (CHC) underwent chemical deacetylation with concentrated NaOH (50% aqueous solution), an approach chosen due to the need for a shorter reaction time. CHC and NaOH solutions were introduced, in a 1:20 (*w*/*v*) ratio, in a laboratory setup consisting of a round bottom flask and refrigerant. The flask was placed on a hot plate, under magnetic stirring. The reaction took place at a temperature of 80 °C and a stirring speed of 400 rpm. Nitrogen was purged into the flask at the beginning of the process (during the first 10 min of the process) to remove any dissolved oxygen. At the end of the synthesis, the samples were rinsed with distilled water until attaining a neutral pH and, finally, vacuum-filtered. The chitosan samples (CCHC) were then dried to constant mass in an oven at 60 °C.

The preparation conditions for the various commercial chitin-derived chitosan samples are given in Table S1 (Supporting Information).

#### 4.2.2. Isolation of Chitin and Synthesis of Chitosan from Shrimp Shell Waste

Usually, the isolation of chitin (CHSH) and the following synthesis of chitosan (CCHSH) from shrimp shell waste is a time-consuming and multi-stage process, which, in our case, involved only two classical steps: deproteinization and deacetylation. Prior to deproteinization, shrimp carcasses were first subjected to a cleaning and drying procedure, involving the cleansing with distilled water (2 × 20 mL/g of shrimp carcasses), covering with NaCl (150 g NaCl/g of shrimp carcasses), and storing in a vessel, under direct sunlight (48 h). Subsequently, the shells were ground in a blender and covered again with NaCl (150 g NaCl/g of shrimp carcasses) for another 24 h in sunlight, followed by washing with distilled water to remove the residual salt until neutral pH and freeze-drying at −55 °C for 24 h to obtain shrimp shell flakes.

Deproteinization involved treating the shrimp shell flakes with an aqueous 1 M NaOH solution (SH:NaOH = 1:15, *w*/*v*) for 24 h at 25 °C, under magnetic stirring (200 rpm). After deproteinization, the samples were washed with distilled water in 2 × 250 mL portions (neutral pH) and vacuum-filtered. The obtained chitin samples (CHSH) were again freeze-dried for approximately 24 h beforehand.

Finally, deproteinized CHSH samples (protein content evaluated by UV-Visible spectroscopy according to Figure S3 (Supporting Information)) were chemically deacetylated with a NaOH 50% solution at a 1:20 ratio (chitin:NaOH), as described in Section 2.2.1, with the mention that the synthesis time was conducted in a single batch for 6 h (Table S1, Supporting Information), as resulted from the optimization of chitin deacetylation.

### 4.3. Determination of Deacetylation Degree (DD)

For this particular measurement, a classical procedure was applied, using titration. In a typical trial, 0.1 g of chitosan was immersed in 20 mL of distilled water under magnetic stirring (900 rpm). The initial pH value was measured with a pH meter, after which 0.2 mL of 0.1 M HCl was added every 10 s and the pH of the solution was measured until the chitosan sample was completely dissolved. The DD was determined using Equation (1), in which case the commercial chitosan, with a known DD of 79%, was used as a reference.

(1) $${\mathrm{DD}}_{n}=\frac{n_{{\mathrm{NH}}_{2},\mathrm{Cn}}\times {\mathrm{DD}}_{\mathrm{CC}}}{n_{{\mathrm{NH}}_{2},\mathrm{CC}}}$$

where DD_CC_ = deacetylation degree of commercial chitosan (79%); $n_{{\mathrm{NH}}_{2}\mathrm{CC}\;=}$ moles of -NH_2_ in 0.1 g of commercial chitosan with DD of 79% (0.000393 moles); $n_{{\mathrm{NH}}_{2},\mathrm{Cn}}$ = moles of -NH_2_ in 0.1 g chitosan calculated by Equations (2) and (3):

(2) $$n_{{\mathrm{NH}}_{2},\mathrm{Cn}}=n_{{\mathrm{NH}}_{2},\mathrm{CC}}+n_{\mathrm{dif}}$$

(3) $$n_{\mathrm{dif}}=(V_{\mathrm{HCl},\mathrm{Cn}}-V_{\mathrm{HCl},\mathrm{CC}})\;\times {\;C}_{M,\mathrm{HCl}}$$

where n_dif_ represents the moles of chitosan samples; V_HCl, Cn_ = volume of HCl 0.1 M necessary to titrate the laboratory-obtained chitosan; V_HCl, CC_ = volume of HCl 0.1 M necessary to titrate the commercial chitosan; C_M, HCl_ = molar concentration of HCl (0.1 M).

In order to determine the degree of deacetylation (DD) by titration, a Jenway 3510 pH meter was used. The volume of HCl required for titration was computed from the pH variation graph as a volume function by determining the inflection point.

### 4.4. Characterization Techniques

Gel Permeation Chromatography (GPC). The molecular weight distributions and the polydispersity indexes were determined on an HPLC 1200 Series with a Refractive Index Detector (RID) from Agilent Technologies equipped with a PLGel Mixed-C (300 × 7.5 mm) Column and isocratic pump. The column was calibrated using polyethylene oxide (PEO) EasiCal standards in the 106–1,522,000 g/mol range of molecular weight (Mn). To this end, chitosan samples were dissolute in an aqueous acetic acid solution (5% AcOH in ultrapure water, or 10% for CCHSH), filtered, and injected at 25 °C at a flow rate of 1 mL/min.

Fourier Transform Infrared (FTIR) spectra of the samples were recorded on a Nicolet™ Summit PRO FTIR Spectrometer (Thermo Fisher Scientific, Waltham, MA, USA) by acquiring 16 scans with 4 cm^−1^ resolution in the 4000–500 cm^−1^ region.

X-ray diffraction (XRD) patterns were collected with a Rigaku SmartLab equipment, operated at 45 kV and 200 mA, with Cu Kα radiation (wavelength λ = 0.1541 nm) in parallel beam configuration (2θ/θ scan mode). The scanned range was 2 h = 2–60°, with a scan rate of 1°/ min. According to the literature [32], the crystallinity of chitin and chitosan may be assessed using Equations (4) and (5):

(4) $$\;{\mathrm{CrI}}_{020}=\left(I_{020}-\frac{I_{\mathrm{am}}}{I_{020}}\right)\times 100$$

(5) $${\mathrm{CrI}}_{110}=\left(I_{110}-\frac{I_{\mathrm{am}}}{I_{110}}\right)\times 100$$

where I_020_ is the maximum intensity of the crystalline peak from the (020) lattice diffraction, I_110_ is the maximum intensity of the crystalline peak from the (110) lattice diffraction, and I_am_ is the intensity of amorphous diffraction at 2θ = 12.6°.

Thermogravimetric Analysis/Differential Thermogravimetry (TGA/DTG) results were recorded using a TA Instruments Q500 device. About 3 mg of sample was heated from 20 to 700 °C at a heating rate of 10 °C/min under a nitrogen flow (20 mL/min).

Brunauer–Emmett–Teller (BET) and Barrett–Joyner–Halenda (BJH) Methods. BET surface area and pore measurements for raw and obtained materials were carried out by nitrogen adsorption using a NOVA 2200 analyzer, a product by Quantachrome Instruments. Nitrogen sorption isotherms at −196 °C were recorded after all the samples were outgassed at 60 °C for 4 h under vacuum prior to N_2_ adsorption.

The rheological study was performed using the hybrid rheometer Discovery HR 20 (TA Instruments, New Castle, DE, USA) at a constant temperature of 25 °C using in the oscillating module a plate-type geometry with a diameter of 40 mm operated at an angular frequency of 0.1–200 rad/s and a speed shear of 0.1–1000 s^−1^, to determine the complex viscosity (Pa·s). The 10 mL solutions were prepared in a vial under magnetic stirring (900 rpm) at 30 °C by the dissolution of 0.1 g of chitosan in a solvent mixture consisting of HCl and water. For samples CC and CCHC6, a 1:1 (water:HCl) solvents ratio was used, whereas, for the CCHSH sample, the ratio was 2:3, because of its lower solubility.

## Figures and Tables

**Table 1:** DD, molecular weight, and polydispersity index for chitosan synthesized from commercial chitin and shrimp shells.

Sample	DD% ^a^	*M_n_* (g/mol)		*PI* ^b^		Area (%)	
		(Peak 1)	(Peak 2)	(Peak 1)	(Peak 2)	(Peak 1)	(Peak 2)
CC	79.00	2.045 × 10^5^	-	2.39	-	100	-
CCHC4	70.95	6.558 × 10^5^	1.062 × 10^4^	19.47	1.11	90.1	9.9
CCHC4(2)	74.57	7.837 × 10^5^	1.224 × 10^4^	22.53	1.02	98.1	1.9
CCHC6	78.19	5.729 × 10^5^	1.158 × 10^4^	26.26	1.09	92.7	7.3
CCHSH	75.38	1.655 × 10^6^	9.059 × 10^3^	6.19	1.39	29.7	70.3

^a^ Determined by titration according to Equation (1). ^b^ Polydispersity index of the synthesised chitosan samples, *PI* = *M_w_/M_n_*, given by GPC.

**Table 2:** Content in crystalline phase (determined from XRD), and total mass loss and maximum degradation temperatures, T_max_ (investigated by thermal analysis), for chitosan and chitin samples.

Sample	*CRI_020_*	*CRI_110_*	Total Mass Loss (%)	T_max_ (°C)		
				1	2	3
SH	55.55	55.55	65.57	56.18	300 (shoulder)/352.93	662.59
CHSH	61.26	57.00	76.64	53.91	362.08	678.34
CHC	71.23	78.78	99.50	33.4	367.46	-
CC	37.03	66.00	68.93	44.57	287.84	-
CCHC6	53.08	62.00	68.35	44.82	288.38	-
CCHSH	59.42	44.55	55.02	45.77	305.40	691.92

**Table 3:** BET surface area and pore analysis of tested materials.

Sample	BET Surface Area (m^2^ g^−1^)	Pore Surface Area (m^2^ g^−1^)	Maximum Pore Diameter ^a^ (nm)	Micropore Volume ^b^ (cm^3^ g^−1^)
SH	3.364	3.989	4.15	0.0051
CHSH	9.805	13.860	3.97	0.0224
CHC	6.875	7.657	3.17	0.0142
CC	0.279	0.899	4.54	0.0011
CCHSH	7.817	11.16	4.15	0.0415
CCHC6	2.164	2.436	4.54	0.0030

^a^ Calculated by BJH method. ^b^ Measured at P/P0 = 0.99.

## Data Availability

Not applicable.

## Supplementary Materials

The following supporting information can be downloaded at: https://www.mdpi.com/article/10.3390/gels8110742/s1, Figure S1: Chitosan pH variance as a function of 0.1 M HCl volume for CCHC4 (a), CCHC4(2) (b), CCHC6 (c), CCHSH (d), and CC I chitosan samples; Figure S2: Molecular weight distribution curves for synthesized CCHC4, CCHC4(2), CCHC6, CCHSH, and CC chitosan samples; Figure S3: UV-Vis analysis of the washing water of chitin sample after its deproteinization; Table S1: Chitosan samples from commercial chitin and shrimp shells and their preparation conditions.
