# Supplementary material for: A Top-Down Procedure for Synthesizing Calcium Carbonate-Enriched Chitosan from Shrimp Shell Wastes
Source: Gels. 2022 Nov 15;8(11):742. doi: 10.3390/gels8110742 (PMC9690297; doi:10.3390/gels8110742)
Supplement: Supplementary file 1 [file gels-08-00742-s001.zip › gels-1999768-supplementary.pdf]

Supplemental Material

for

A top-down procedure for synthesising calcium carbonate enriched -chitosan from shrimp shell wastes

Andreea Miron[1,2†], Andrei Sarbu[1†], Anamaria Zaharia[1], Teodor Sandu[1], Radu Claudiu Fierascu[1], Ana-Lorena Neagu[1], Horia Iovu[2], Anita-Laura Chiriac[1*], Tanta-Verona Iordache[1]

[1]National Institute for Research & Development in Chemistry and Petrochemistry ICECHIM
[2]Advanced Polymer Materials Group, University POLITEHNICA of Bucharest,1-7 Gh. Polizu Street, 011061, Bucharest, Romania
*Correspondence: *E-mail address:* anita-laura.radu@icechim.ro ((+40)723599783), tanta-verona.iordache@icechim.ro ((+40)755159896)
[†]These authors contributed equally to this work.

Manuscript Section 2.1. and 2.2

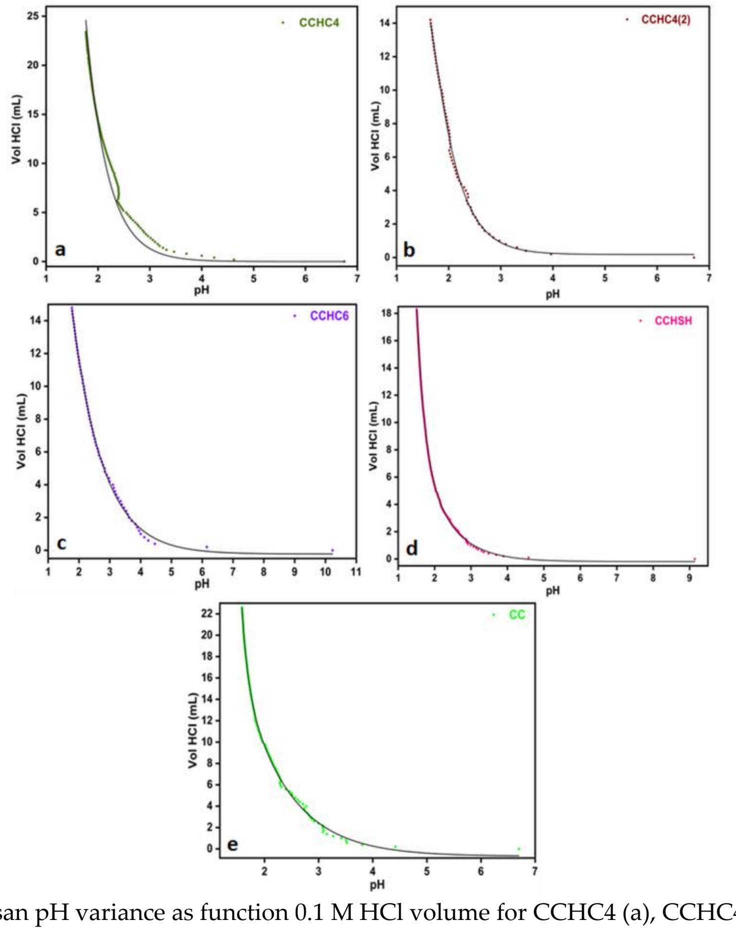

Figure S1. Chitosan pH variance as function 0.1 M HCl volume for CCHC4 (a), CCHC4(2) (b) and for CCHC6 (c), CCHSH (d) and CC (e) chitosan samples.

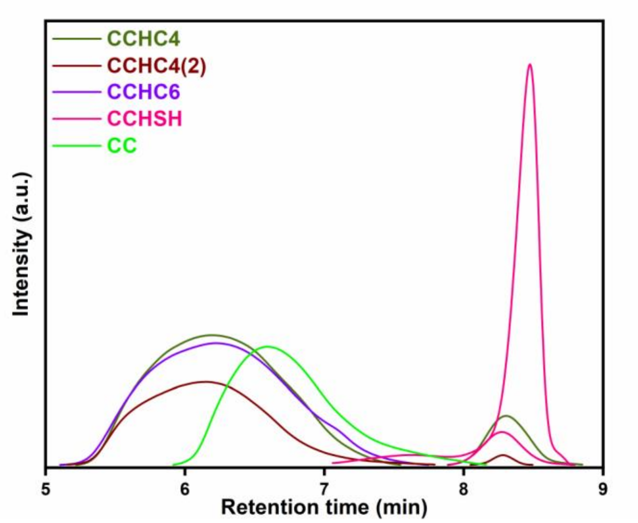

Figure S2. Molecular weight distribution curves for synthesized CCHC4, CCHC4(2), CCHC6, CCHSH and CC chitosan samples.

Manuscript Section 4.2. Synthesis of chitosan

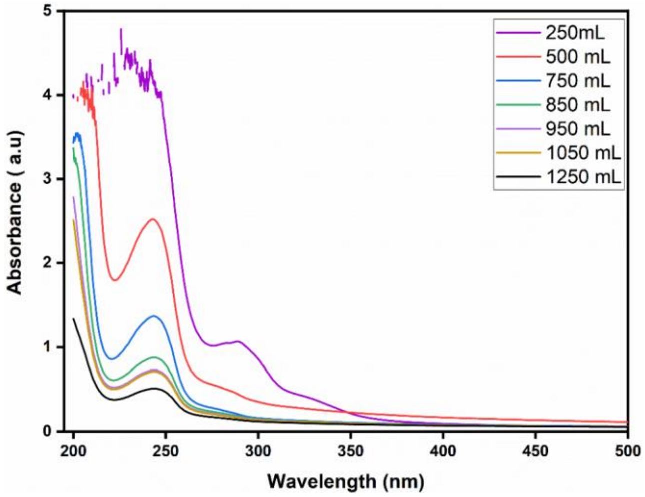

Figure S3. UV-Vis analysis of the washing water of chitin sample after its deproteinization.

The content of proteins after washing was monitored by Ultraviolet-Visible Spectroscopy (UV-Vis) with an Evolution 260 BIO spectrophotometer (Thermo Scientific). The release of proteins in the supernatant was followed by the measurement of the absorbance at λ = 280 nm, characteristic of the tryptophan residues found in the protein composition. Removal of the associated proteins is an essential step in the polysaccharide purification process [1]. In our studies, after a first step, which is very fast, we may consider that, even after 24 h of treatment in 1 M NaOH, at ambient temperature, the deproteinization was not completely achieved. For this reason, we investigated this process by UV-vis analysis. Figure S4 depicts the UV-Vis spectra of washing waters of chitin sample after completion of the deproteinization process of shrimp shell waste as a result of repeated washing cycles. A characteristic peak of proteins was recorded at 245 nm [2] and at 280 nm (specific for the presence of tryptophan residues) whose intensity was lower after washing. Thus, it may be concluded that the deproteinization

step was successfully accomplished. Proteins were removed quantitatively from the shrimp shells and chitin was obtained successfully [2,3].

Table S1. Chitosan samples from commercial chitin and shrimp shells and their preparation conditions

Sample	NaOH sol. (%)	Deacetylation cycles	Reaction time for the first deacetylation cycle (h)	Reaction time for the second deacetylation cycle (h)
CCHC4	50	1	4	0
CCHC4(2)	50	2	4	2
CCHC6	50	1	6	0
CCHSH	50	1	6	0

Reference

1. Teng, W.L.; Khor, E.; Tan, T.K.; Lim, L.Y.; Tan, S.C. Concurrent production of chitin from shrimp shells and fungi. *Carbohydr. Res.* **2001**, *332(3)*, 305–316. doi:10.1016/s0008-6215(01)00084-2

2. Antosiewicz, J. M.; Shugar, D. UV–Vis spectroscopy of tyrosine side-groups in studies of protein structure. Part 2: selected applications. *Biophys.Rev.* **2016**, *8(2)*, 163–177. doi:10.1007/s12551-016-0197-7

3. Kumirska, J.; Czerwicka, M.; Kaczyński, Z.; Bychowska, A.; Brzozowski, K.; Thöming, J.; Stepnowski, P. Application of Spectroscopic Methods for Structural Analysis of Chitin and Chitosan. *Mar. Drugs.* **2010**, *8(5)*, 1567–1636. doi:10.3390/md8051567
